# Targeted Exome Sequencing Integrated with Clinicopathological Information Reveals Novel and Rare Mutations in Atypical, Suspected and Unknown Cases of Alport Syndrome or Proteinuria

**DOI:** 10.1371/journal.pone.0076360

**Published:** 2013-10-10

**Authors:** Rajshekhar Chatterjee, Mary Hoffman, Paul Cliften, Surya Seshan, Helen Liapis, Sanjay Jain

**Affiliations:** 1 Department of Internal Medicine (Renal division), Washington University School of Medicine, St. Louis, Missouri, United States of America; 2 Department of Genetics, Washington University School of Medicine, St. Louis, Missouri, United States of America; 3 Department of Pathology and Immunology, Washington University School of Medicine, St. Louis, Missouri, United States of America; 4 Department of Pathology, Weill Medical College, Cornell University, New York, New York, United States of America; Fondazione IRCCS Ospedale Maggiore Policlinico & Fondazione D’Amico per la Ricerca sulle Malattie Renali, Italy

## Abstract

We applied customized targeted next-generation exome sequencing (NGS) to determine if mutations in genes associated with renal malformations, Alport syndrome (AS) or nephrotic syndrome are a potential cause of renal abnormalities in patients with equivocal or atypical presentation. We first sequenced 4,041 exons representing 292 kidney disease genes in a Caucasian woman with a history of congenital vesicoureteral reflux (VUR), recurrent urinary tract infections and hydronephrosis who presented with nephrotic range proteinuria at the age of 45. Her biopsy was remarkable for focal segmental glomerulosclerosis (FSGS), a potential complication of longstanding VUR. She had no family history of renal disease. Her proteinuria improved initially, however, several years later she presented with worsening proteinuria and microhematuria. NGS analysis revealed two deleterious *COL4A3* mutations, one novel and the other previously reported in AS, and a novel deleterious *SALL2* mutation, a gene linked to renal malformations. Pedigree analysis confirmed that *COL4A3* mutations were nonallelic and compound heterozygous. The genomic results in conjunction with subsequent abnormal electron microscopy, Collagen IV minor chain immunohistochemistry and progressive sensorineural hearing loss confirmed AS. We then modified our NGS approach to enable more efficient discovery of variants associated with AS or a subset of FSGS by multiplexing targeted exome sequencing of 19 genes associated with AS or FSGS in 14 patients. Using this approach, we found novel or known *COL4A3* or *COL4A5* mutations in a subset of patients with clinically diagnosed or suspected AS, *APOL1* variants associated with FSGS in African Americans and novel mutations in genes associated with nephrotic syndrome. These studies demonstrate the successful application of targeted capture-based exome sequencing to simultaneously evaluate genetic variations in many genes in patients with complex renal phenotypes and provide insights into etiology of conditions with equivocal clinical and pathologic presentations.

## Introduction

Glomerular dysfunction is the most common cause of end stage renal disease [1], [2]. Damage to the glomeruli can manifest in a number of ways including proteinuria, hematuria and hypertension [1], [3], [4], [5], [6], [7]. Many glomerular diseases are categorized according to well-defined histopathological patterns. For example, focal segmental glomerulosclerosis (FSGS) pattern in a biopsy can be caused by different etiologies including primary or secondary glomerular disease or reflux due to lower urinary tract malformations [8]. Newer genomic technologies including next-generation sequencing (NGS) are rapidly evolving and may provide new insights into disease pathogenesis, diagnosis, genetics or prognosis, explain histopathological findings and potentially guide therapy. However, their application to kidney disease has been limited.

We first analyzed exomes of several hundred genes using a targeted customized exome sequencing approach in a patient with a history of vesicoureteral reflux (VUR) who presented with proteinuria and FSGS on renal biopsy. We identified two deleterious nonallelic *COL4A3* mutations, one novel and the other previously reported in an Alport syndrome (AS) patient. We further identified a deleterious mutation in *SALL2*, a gene important in early kidney development. Pedigree analysis confirmed that the *COL4A3* mutations were compound heterozygous and subsequent clinical work-up confirmed dual disease, Alport syndrome and VUR. We then applied a modified targeted exome sequencing by multiplexing 19 genes and 14 patients with either well established or clinically suspected AS or FSGS to determine potential genetic cause of their diseases. Several novel and previously known *COL4A* mutations in AS, *APOL1 G2* deletion variants in some FSGS patients and new coexisting gene mutations in proteinuria related genes (*LAMA5*) were identified. These studies are among the first to apply Next-gen targeted-capture exome sequencing approach in delineating genetic basis for atypical and complex renal diseases presenting as renal malformations and/or glomerular diseases.

## Materials and Methods

### Human Studies

Human studies were conducted according to protocols approved by the Washington University human research protection office. Patient recruitment, specimen collection and processing were done by the Washington University Kidney Translational Research Core. Samples from 15 patients were used (14 glomerular disease, 1 glomerular and renal malformation).

### Gene Selection

**Figure 1:**
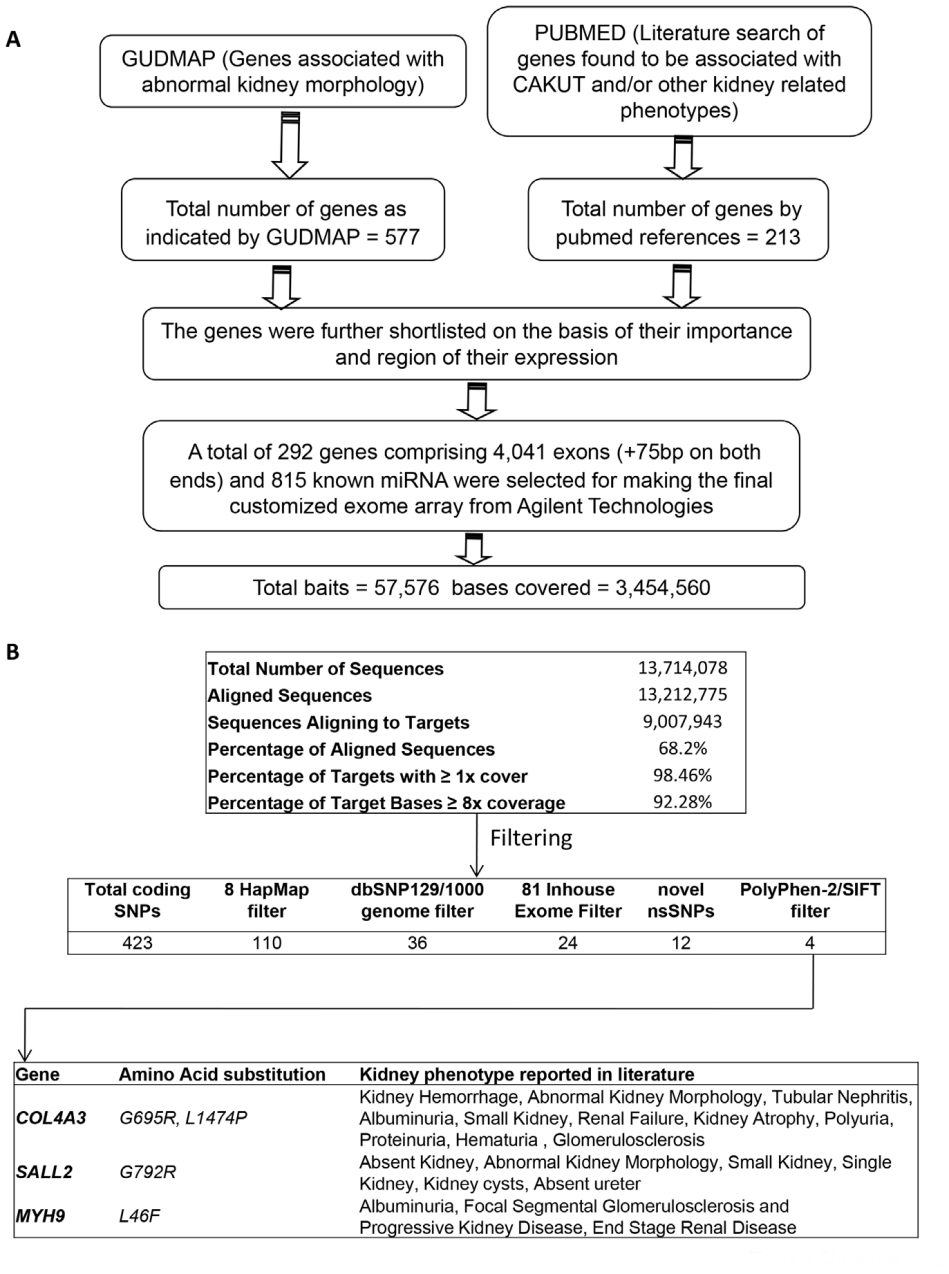
Overview of the targeted exome sequencing pipeline and filtering strategy applied to patient with VUR and proteinuria. (A) Criteria for gene selection for developing a custom targeted renal disease exome array. (B) Custom-exome capture sequencing metrics from the patient 1184405 and filtering approach used to identify four novel mutations in three kidney disease genes.

The genes selected for this study resulted from searches of public databases such as PUBMED and GUDMAP (http://www.gudmap.org/) and abstracts as of 2010 (Figure 1a). Briefly, search terms included genes associated with CAKUT and/or other kidney related phenotypes such as renal failure, abnormal kidney development, ectopic kidney, atrophic kidney, single kidney, no kidney, and kidney disease. The gene lists were further curated based on their expression patterns, functional significance in disease models and mutations in human kidney disease. A total of 292 genes comprising 4,041 exons were selected for the sequencing studies (Table S1). The genomic intervals for the shortlisted genes were selected using GRch37/hg19 version of the assembled human genome. The UCSC genome browser (http://genome.ucsc.edu/cgi-bin/hgTables?org=Human&db= hg19&hgsid = 192997943&hgta_doMainPage = 1) was used to obtain the start and end sites of each exon including known isoforms and 75 bp of flanking regions were additionally included to cover the exon-intron boundaries and the splice junctions. In some cases, 10 Kb promoter regions were also included; however, this analysis was focused on exons and flanking sequences.

### Custom Bait Library Generation

Bait generation and library preparation were done using the eArray tool provided by the manufacturer (Agilent technologies, (https://earray.chem.agilent.com/earray)). The baits, 120 bp each, were generated for Illumina end sequencing technology with bait tiling frequency of 2X using optimized parameters. The failed baits (632) were redesigned with the repeatmasker filter turned off. We also included baits for sequencing 815 miRNA that were already predesigned by Agilent. Library was created using Library preparation tool of Agilent for paired end sequencing. The final library generated consisted of 57,576 total functional baits that cover 3,454,560 bp of the genome. For the screening of mutations in patients with proteinuria or AS multiplexed targeted exome capture and NGS was done on a set of 19 genes (Supplemental Table S3) known or predicted to be associated with AS or proteinuria. Gene selections and library preparation were same as described above.

### DNA Extraction, Library Preparation, Next-generation Sequencing and Data Filtering

Genomic DNA was obtained from the Washington University Kidney Translational Research Core. Briefly, genomic DNA was isolated from peripheral blood leukocytes using QIAamp DNA blood mini kit (Cat No 51106) according to manufacturer’s instructions. Genomic DNA for relatives was isolated from buccal cells using an over the counter mouthwash (Scope). For the patients with AS and CAKUT, library preparation targeted capture against the baits and NGS using GAIIx sequencer (Illumina) was done by the Genome Technology Access Center core facilities.

Novoalign (http://www.novocraft.com) was used for alignment of the sequencing reads and variant calling was done using SAM tools [9] as described before [10]. SNP calls were filtered against the dbSNP129 database, in-house exome data and prioritized according to importance with publicly available software tools (Figure 1b). The effect of amino acid substitution on protein function was predicted with Sorting Intolerant from Tolerant (SIFT, http://sift.jcvi.org/) and Polymorphism Phenotyping v2 (PolyPhen-2, http://genetics.bwh.harvard.edu/pph2/) [11]. Controlled access to sequencing data are available to investigators authorized to conduct human studies. Genotype data will be deposited at the European Genome-phenome Archive (EGA, http://www.ebi.ac.uk/ega/), which is hosted by the EBI, under accession number EGAS00001000560.

### Sanger Sequencing

Novel or rare variants identified from exome sequencing in the patient with VUR and proteinuria were validated using Sanger sequencing. All primers used are shown in Supplemental Table S2. Sanger sequencing was performed in the tissue procurement and molecular phenotyping core at the Washington University. The data were analyzed using DNASTAR software (http://www.dnastar.com/).

### Histopathology

Histopathological results were obtained from the Pathology archives of Washington University School of Medicine in St. Louis and Cornell University. These data were part of routine care involving light microscopy, immunohistochemistry and electron microscopy of renal biopsies.

### Sanger Sequencing Validation of the *APOL1* Deletion in African American Focal Segmental Glomerulosclerosis (FSGS) Patients

To confirm the APOL1deletion variation (rs71785313), also known as G2, in the two African American (AA) FSGS patients, Sanger sequencing was performed using the APOL1_I/D_rs71785313 primer set (Supplemental Table S2). The same primer set was used to determine the allelic frequency of the deletion in a set of African-American FSGS patients and unrelated controls.

## Results

### Clinical Phenotype of a Patient with Unexplained Proteinuria

We enrolled a Caucasian woman (patient 1184405) whose past medical history is significant for vesicoureteral reflux (VUR) diagnosed at age two, several urinary tract infections and hydronephrosis. At age 45 she presented with nephrotic range proteinuria and hypertension but no evidence of hematuria. Her kidney biopsy showed histopathologic changes that suggested focal segmental glomerulosclerosis (FSGS), however, immunohistochemical and electron microscopy studies were inconclusive. She has no family history of renal disease. Her proteinuria initially improved on oral steroids but later required additional immunosuppressants due to worsening creatinine; proteinuria range was 2+ to 3+. It was unclear from the clinical course if the patient had primary or secondary FSGS, for example, due to VUR or another disease. At age 48 she presented with worsening proteinuria, her repeat biopsy showed FSGS, and the electron microscopy findings were suggestive of hereditary nephritis. The patient consented to participate in studies to investigate potential molecular-genetic causes of her kidney disease that may help clarify or provide insights into the underlying etiology of her renal dysfunction.

### Deleterious Mutations in *COL4A3, MYH9* and *SALL2* in the Patient 1184405

**Figure 2:**
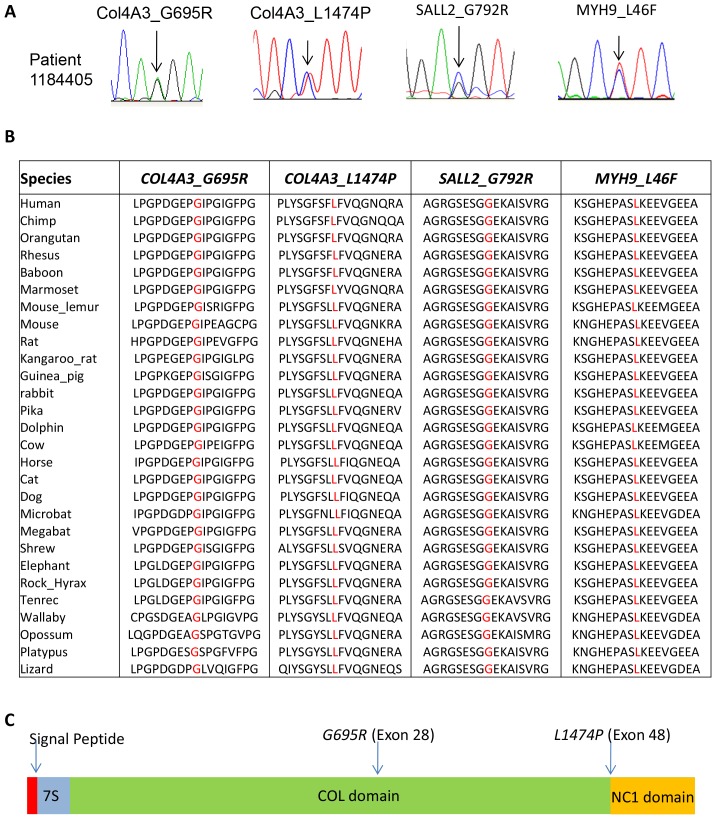
Validation and evolutionary conservation of the identified mutations in the patient with VUR and proteinuria. (A) Sanger sequencing analysis confirms the novel *COL4A3* mutations, *COL4A3_G/A_G695R* and *COL4A3_T/C_L1474P*, the *SALL2_G/C_G792R* and the *MYH9_C/T_L46F* mutations in the patient 1184405. (B) Multi species alignment shows that both the *COL4A3* mutation reference amino acids *Col4A3_ 695_G* and *Col4A3_1474_L*, the *SALL2_G792R* and the *MYH9_L46F* are highly conserved among species. (C) Schematic representation of Human *COL4A3* gene with the discovered mutations in patient 1184405. The domains are: signal peptide domain (red), the N terminal 7S domain (blue), the central triple helix collagenous (COL) domain (green) and the carboxy-terminal non-collagenous (NC1) domain (yellow).

To identify gene defects that may uncover potential cause of CAKUT or proteinuria that lead to kidney dysfunction in the patient 1184405 we sequenced the coding region and exon-intron splice junctions of 292 genes most likely associated with renal anomalies or glomerular disease using NGS (see methods). Sequencing results showed high depth of coverage with more than 90% of the targeted region sequenced at a depth of 8-fold or greater (Figure 1b). To prioritize identification of mutations that are the most deleterious we adopted a filtering strategy to focus on non-synonymous novel deleterious variants using a combination of filtering against SNP databases, in-house unrelated non-kidney disease exomes and bioinformatic software tools Polyphen2 and SIFT (Figure 1b) [10]. We discovered four novel non-synonymous mutations: *COL4A3_G695R*, *COL4A3_L1474P*, *MYH9_L46F* and *SALL2_G792R* that were all predicted to be damaging (Figure 1b). All four mutations were confirmed by Sanger sequencing (Figure 2a). Bioinformatic analysis revealed that the mutated residues were conserved across species (Figure 2b). The *COL4A3* mutations were in a highly conserved glycine rich region and in an important functional domain (Figure 2c).

### Pedigree Analysis of the Four Deleterious Mutations in Patient 1184405

**Figure 3:**
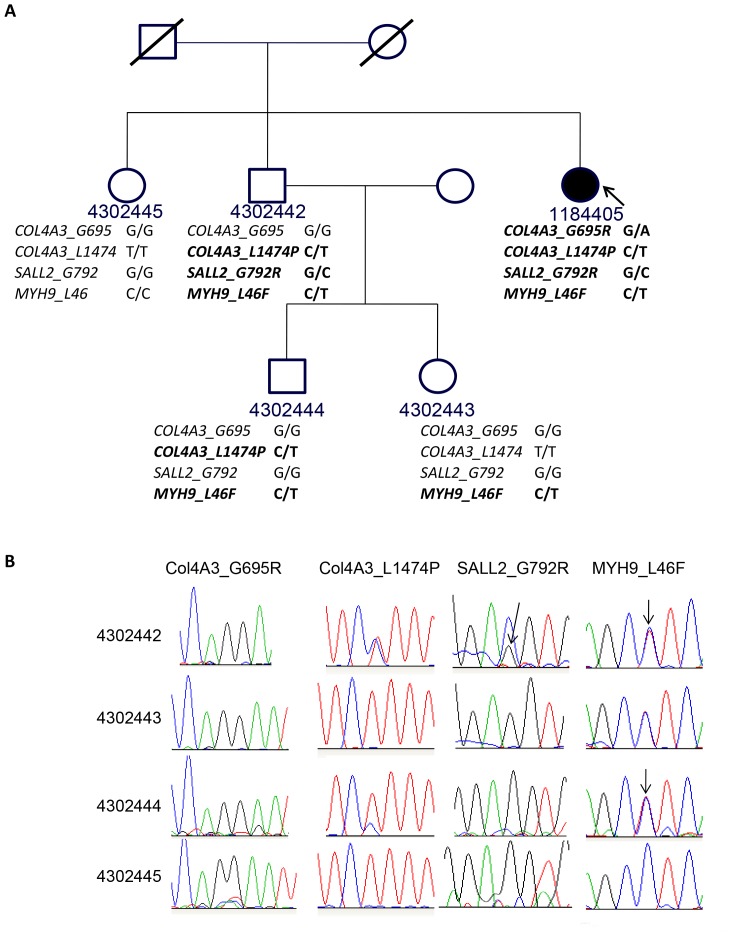
Pedigree analysis of the index patient with proetinuria and VUR. (A) Pedigree of the family of the index patient (1184405, arrow) shows presence of *Col4A3_T/C_L1474P* variant in one unaffected brother (4302442) and nephew (4302444), but absent in the unaffected sister (4302445) and niece (4302443); while the other damaging variant *Col4A3_G/A_G695R* is present only in the patient. The *SALL2* deleterious mutation *SALL2_G/C_G792R* is present in the patient and one unaffected brother (4302442) while the *MYH9_C/T_L46F* mutation is present in all but one affected family member (4302445). (B) Sanger sequencing analysis confirms the status of the novel *COL4A3* mutations *Col4A3_G/A_G695R* and *Col4A3_C/T_L1474P*, and the *SALL2_G/C_G792R* and *MYH9_C/T_L46F* mutations in the relatives of patient 1184405. The *Col4A3_T/C_L1474P* heterozygous variant was present in one unaffected brother (4302442) and nephew (4302444), but absent the unaffected sister (4302445) and niece (430443). The mutation *Col4A3_G/A_G695R* was absent in all other family members. The *SALL2_G/C_G792R* mutation was present only in the unaffected brother 4302442 while the *MYH9_C/T_L46F mutation* was more common and was present in everyone except the unaffected sister 4302445.

The presence of *COL4A3* mutations suggested Alport syndrome in this patient as a likely cause of proteinuria (see below). We next performed studies to determine the inheritance pattern of the deleterious mutations found in patient 118405. Consent was obtained from the available family members (Figure 3). Sanger sequencing was performed on their genomic DNA to detect *COL4A3-G695R* and *COL4A3-L1474P* mutations. None of the family members harbored both *COL4A3* mutations suggesting the possibility that they are on separate alleles in the index patient (Figure 3a,b). The *COL4A3-L1474P* mutation was found in an unaffected brother and his son with unknown disease status. The *COL4A3-G695R* mutation was not present in any of the family members studied indicating the possibility of a de novo change. Mutation analysis of *SALL2* and *MYH9* was also performed by Sanger sequencing in all the available family members and none of the family members harbored all four mutations (Figure 3a and 3b).

### Immunohistochemistry and Electron Microscopy Confirm Collagen Defects

**Figure 4:**
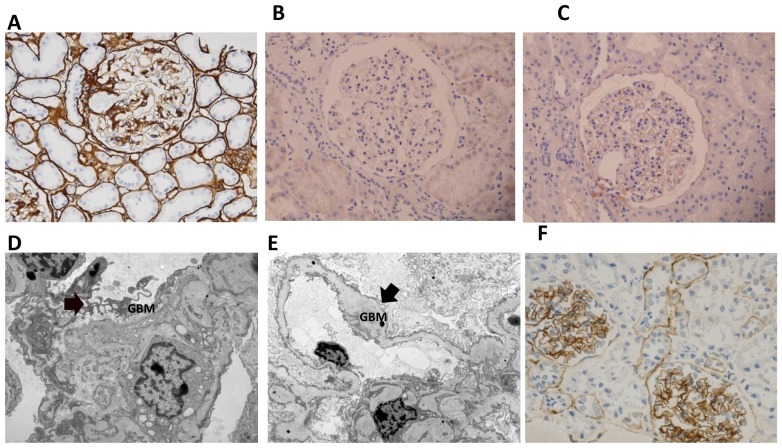
Results of immunostaining with antibodies against α1, α3, and α5 chains of type IV collagen in the AS/VUR patient. (A) ColIVα1 expression is strong and consistent in the basement membranes (BM) of the glomeruli and the tubules; typically it is absent in adult glomeruli. Images show absent α3IV (B) and weak α5IV (C) staining in the glomerular BMs. (D&E) Electron micrograph from this patient shows segmental glomerular basement membrane (GBM) lamellation and outpouching (D arrow) and irregular thickening (E arrow) of the GBM characteristic of AS. COL4α3 immunostaining in control shows the normal tubular and capillary loop distribution for comparison (F).

At age 52 the patient [1184405] presented with proteinuria (3+) and hematuria (1+) and reported hearing loss of two years duration. A kidney biopsy was performed to confirm hereditary nephritis (AS) as a cause of her worsening renal dysfunction. Light microscopy was remarkable for FSGS. We performed COL4α1, COL4α3 and COL4α5 immunohistochemistry and found strong COL4α1 immunoreactivity in the glomerular capillary loops, and tubular basement membrane, weak COL4α5 in glomerular capillary loops and basement membrane of distal tubules and no COL4α3 immunoreactivity (Figure 4). EM showed glomerular basement membrane thickening and laminations alternating with areas of thinning, findings consistent with AS.

### Custom Next-generation Sequencing of Candidate Proteinuria Genes Simultaneously in Several Patients (Multi-indexed) Provides Genetic Evidence for Cause of Alports or FSGS

We next extended our above analysis to a modified NGS approach to identify mutations in a selected group of genes associated with proteinuria in patients with clinically diagnosed Alports or FSGS or cases in which diagnosis was equivocal (see Table 1 for patient characteristics). We sequenced coding region of a set of 19 genes (Supplementary Table 3) in nine patients with a clinical diagnosis of hereditary nephritis or AS and five FSGS patients. One of the patients with FSGS diagnosis (07-0430-01590) is a relative of another patient with AS (07-0430-02009). As indicated in Table 1, some of these patients had established AS based on family history or pathological findings while in others there was a family history of kidney disease but no pathologically or genetically confirmed AS. The FSGS patients were selected to serve as negative controls for *COL4* mutations and to determine if variations in genes associated with proteinuria may explain the etiology in some of these patients. We applied a multiindexing approach where each patient’s DNA was coded with a unique tag (index) so the targeted exomes from all the patients could be simultaneously sequenced. NGS metrics revealed greater than 200-fold average coverage and 95% of the region of interest was covered at a depth of 40X or greater (Supplementary table 4). Using the same filtering approach as for the patient with Alports and VUR above we found *COL4A3*, *COL4A4* and/or *COL4A5* novel or rare mutations in 6 out of 9 unrelated patients with known or equivocal AS and novel *LAMA5* mutations in 3 out of 4 FSGS patients (Table 1). Two relatives (07-0430-02009 and 07-0430-01590) were included and the same *COL4A5-G1161E* mutation was independently identified providing credence to our approach. Among these, patient 07-0430-01590 is the grandmother of 07-0430-02009 and did not previously have AS diagnosis and is clinically phenotyped as FSGS. Based on bioinformatics predictions a number of these variants were predicted to be probably damaging using the PolyPhen 2 software (Table 1). No small indels or exon deletions were present in the Collagen *4A3, A4 or A5* genes. Visual inspection of the mutations identified by SAMtools confirmed the base calls made. We also detected a previously reported 6 bp deletion in *APOL1* (G2 variant) that is associated with the development of FSGS in African Americans [12] and verified this variant using Sanger sequencing.

**Table 1:** Clinical characteristics of patients that underwent validation of custom multindexed targeted exome sequencing of 19 genes.

Participant ID	Diagnosis	Age enrolled	Age diagnosed	Gender	Race	Novel deleterious Mutations	PolyPhen-2scores ofNovelMutations	RareDeleteriousMutations(MAF<0.05)	Family History	Biopsy (initial clinical presentation)
07-0430-01196	Alport’s syndrome/ESRD	47	10	F	C	*COL4A5-G1030S*	1		father, sister andpaternal uncle allon Hemodialysis. Fatherdiagnosed for Alport’s.Two sons withpresumed Alport’s	biospy at age 10, inconclusive (proteinuria)
07-0430-01724	Alport’s sydrome	8	4	M	C			rs1800516	3 brother withAlport’s Syndrome	skin negative for COL4A5 (hematuria)
*07-0430-02009*	horseshoe kidney/Crohn’s/alport’s	16	6	F	C	*COL4A5-G1161E*	1	rs13027659	Grandmother07-0430-00730 hasESRD/FSGS	Alport Syndrome (GN)
*07-0430-01590*	ESRD, Grandmother of07-0430-2009	53	11	F	C	*COL4A5-G1161E,* *NPHS1-N188I*	1, 0.57		maternal uncle and 2cousins died of renalfailure	FSGS (proteinuria)
07-0430-00730	Hereditary nephritis	70	44	M	C				brother with renal transplant	no biopsy (ESRD)
07-0430-01161	Hereditary nephritis	71	56	F	C				multiple familymembers with kidney transplants	no biopsy (ESRD)
07-0430-01559	Hereditary nephritis	60	48	F	C				likely secondary FSGS	no biopsy (Cr 1.8)
07-0430-01722	Hereditary nephritis	75	60	M	C	*COL4A3-S1147F,* *COL4A4-P1587R*	0.655, 0.913		brother and sister withESRD due to presumedhereditary nephritis	no biopsy (ESRD)
07-0430-02027	hereditary nephritis	46	35	M	C			rs1800516	aunt on Hemodialysisand cousin with kidneydisease	no biopsy (ESRD)
07-0430-00597	alport’s syndrome	19	6	M	C				mother withinterrupted COL4A5 staining inskin biopsy	skin negative for COL4A5 (proteinuria)
07-0430-00016	FSGS	38	27	F	AA	*LAMA5-S1469A,* *LAMA5-V2440I*	0.99, 0.65	rs34728338, rs36121515, rs80109666	Father with CKD,diabetes	FSGS (proteinuria)
07-0430-00033	persumed FSGS	62	20	M	AA	*PLCE1-L2173R,* *LAMA5-G3685R*	0.999, 1.0		no family history	no biopsy (inc Cr, HTN)
07-0430-00048	persumed FSGS	45	33	M	C	*CAPN12-R229C,* *LAMA5-E2908V*	1.0, 0.81		no family history	normal COL4A5 (HTN)
07-0430-00099	persumed FSGS	44	32	M	AA	*ACTN4-V801M*	0.013		Father with ESRD,presumed to haveDiabetes Mellitus	biospy inconclusive (proteinuria)

Participants in italics are relatives in which the same mutation was found.

## Discussion

We applied targeted custom exome capture and next-generation sequencing and identified known and/or novel variants in patients with complex kidney disease or where clinicopathological and genetic correlations were discordant. We first applied our strategy to a 54 year old patient with congenital VUR who presented with heavy proteinuria and diagnosis of FSGS. We discovered *COL4A3* mutations that in conjunction with subsequent clinical, family and pathological data undoubtedly identify her as an index case of atypical presentation of AS. We also identified a deleterious *SALL2* mutation as a potential cause of her VUR and an unexpected novel *MYH9* mutation in the same patient. These results demonstrated the power of targeted exome sequencing on selected genes simultaneously to help understand a complex kidney phenotype in a patient and diagnose AS in the absence of typical symptoms or family history. We further showed that this approach is suitable for screening for mutations in selected genes that are associated with glomerular disorders in patients suspected to have AS or FSGS clinically, or detection of G1 or G2 *APOL1* variants in African Americans. The studies suggest that sequencing of a defined set of genes in patients with glomerular diseases or CAKUT may help in early diagnosis and management particularly in complex cases.

We selected several hundred genes important in kidney development and glomerular diseases to identify mutations that may provide insights into the presence of a congenital anomaly (VUR, hydronephrosis) and proteinuria in a patient where the etiology of either entity was unclear since FSGS pattern can be observed in obstructive or reflux nephropathy or primary glomerular diseases. Sequencing of these candidate genes most likely associated with kidney defects and proteinuria revealed 2 mutations in *COL4A3* gene, a gene associated with Alport syndrome and *SALL2*, a gene associated with urinary tract development. Genomic studies confirmed AS in the index patient with no family history of kidney disease, late age of onset, heavy proteinuria, and no hematuria on initial presentation. The patient developed hematuria, sensorineural hearing loss and showed pathological findings characteristic of AS several years after the initial onset of proteinuria. Dysfunction of any of the three-collagen IV genes, *COL4A3, COL4A4* and *COL4A5,* disrupts proper heterotrimer formation resulting in failure to deposit normal collagen matrix in the glomerular basement membrane and disruption of the filtration barrier. Absence of *COL4A3* and *COL4A5* immunolabeling in the patient’s biopsy further confirmed that the *COL4A3* mutations are causative. Pedigree analysis indicated that the two *COL4A3* mutations are on different alleles and compound heterozygous. About 15% of AS cases are thought to exhibit autosomal recessive mode of inheritance [13]. Two of this patient’s relatives had *COL4A3-L1474P* mutation. Whether the second *COL4A3-G695R* mutation occurred *de novo* or was inherited is unclear as the samples from the parents were not available. The relatives with the mutations are reportedly healthy indicating that presence of both the mutations is necessary for developing the disease or there could be other modifiers. In this regard, it was interesting that this patient also had a *MYH9-L46F* mutation. Mutations in *MYH9* have been associated with hearing disorders, kidney defects and thrombocytopenia [14], [15]. We found that the patient’s platelet volume was low, but she did not have thrombocytopenia. Since some of the features of AS [16], [17] and patients with *MYH9* mutations overlap, it is possible that this patient disease phenotype is a result of interactions between these two genes. No other family members had all three mutations suggesting that only collectively they are penetrant. While both *SALL2 and MYH9* mutations likely contribute to the complex phenotype in this patient, biological studies using model systems would be needed to support if these are sufficient or necessary for the phenotype observed. Early onset AS is often associated with large exon deletions, nonsense mutations or splice site changes. The patient had late onset of glomerular disease, which is consistent with the missense *COL4A* mutations.

Early diagnosis of AS, a rare disorder with frequency of 1∶50,000, is clinically important for counseling and clinical management. Existing common methods to diagnose AS include DHPLC and Sanger sequencing. However the large gene size and high number of exons in each of the *COL4* family genes (51 in *COL4A5,* 48 in *COL4A4* and 52 in *COL4A3*), and absence of any mutation hotspot poses difficulties in timely diagnosis and is costly for researches interested in delineating pathogenetic mechanisms. Recently Artuso *et al*., applied amplicon based NGS of just the *COL4A3* genes in AS [18]. While this approach is preferable to the older methods, in atypical cases and in cases where pathology is equivocal such as segmental COL4A3, 4 or 5 staining or insufficient material, information on additional genes (several hundred or less than 30) that may be contributing is needed. Our approach is more efficient and economical as the dynamic range of number of genes to be surveyed can be easily scaled and it allows for indexing (see below) where several patients or family members can be simultaneously evaluated.

Our multiplexed NGS experiments were conducted on 14 patient samples with 19 genes commonly associated with AS or FSGS including steroid resistant nephrotic syndrome. The advantage was that all samples could be simultaneously sequenced and analyzed for variations in these genes at an average read depth of 200X thus significantly increasing the efficiency of obtaining sequence data. We find this approach preferable when the focus is evaluation of small number of genes as an underlying cause of a clinical condition. We focused on AS and included patients with well-documented clinical history and diagnosis. We also included some patients with presumed diagnosis of hereditary nephritis or family history of kidney disease. Our customized exome capture array harbored a subset of genes associated with AS and nephrotic syndrome and confirmed known or novel mutations in *COL4A3*, *A4* or *A5* in four of the five AS patients. Some patients did not have established family history of AS. In two of these patients, we successfully identified novel or known variants that explain their disease. The patients in whom we did not identify deleterious *COL4A* mutations suggest either that these patients need reevaluation of clinical diagnosis or there are changes in *COL4A* gene structures that were not identified here. For example, structural changes in the genome and changes in non coding regions will be missed with current method. FSGS patients were included to serve as negative control for AS and to identify any new mutations in FSGS associated genes. The discovery of novel variants in the *LAMA5* gene has not been reported before. Interestingly three out of five FSGS patients had a novel *LAMA5* mutation predicted to be deleterious. *LAMA5* is important in maintaining the glomerular filtration barrier integrity [19]; however to our knowledge it has not been associated with FSGS previously. Two of the FSGS patients also harbor mutations in *PLCE1*, *NPHS1* and *ACTN4* genes respectively. These genes are associated with FSGS [20], [21], [22], [23] and suggesti that these variants contribute to the disease. It is possible that some of these could be neutral or beneficial or deleterious mutations. We have included prediction scores (Table 1), but unequivocal proof of causation versus neutral variations would require efficient biological validation strategies. Our methods also successfully identified variants that are associated with FSGS in African Americans further portraying the versatile application of NGS in diagnoses and discovery of variants associated with AS or FSGS.

The candidate gene capture and NGS approach we applied is robust in identifying deleterious mutations in different kidney diseases and more importantly if these mutations can provide insights into disease process. This is the first application of this approach to a complex kidney disease presentation and AS, and detecting defined variants associated with FSGS or Alports. Our study demonstrates an integrated approach that utilizes biological and clinicopathologic information in screening defined set of genes for gleaning insights into disease etiology using NGS. These strategies should pave the way to clinical sequencing and high throughput mutation discovery studies compared to single gene approaches that are now inefficient and costly.

## Supporting Information

Table S1 List of 292 candidate genes shortlisted for exome sequencing studies in the patient with VUR and proteinuria.
(XLS)

Table S2 List of primers for validation of COL4A3, SALL2, MYH9 and APOL1 gene variations using Sanger sequencing.
(XLS)

Table S3 List of 19 candidate genes shortlisted for custom multiindexed exome sequencing.able.
(XLSX)

Table S4 Coverage metrics for 19 gene custom exomes nex-gen sequencing in patients with suspected Alports.
(XLSX)
